# A Case of Extra-Uterine Pregnancy

**Published:** 1878-02

**Authors:** Wm. H. Byford


### EXAMINER.

Vol. XXXVI.—FEBRUARY, 1878.—No. 2.

### Original Communications.

### A CASE OF EXTRA-UTERINE PREGNANCY.

### A CLINICAL LECTURE,

By Wm. H. Byford, M. D.

*(Delivered at the Mercy Hospital, and reported by C. D. Jones, M. D.)*

*Gentlemen:* The case I present you to-day is one of supposed extra-uterine pregnancy.

Dr. Charles Cowles, of Barraboo, Wisconsin, brings the patient to the Mercy hospital with the hope that something may yet be done to save her life. Dr. Cowles is an intelligent and experienced physician, and gives the following clear and interesting narrative of the circumstances of the case up to this time:

Mrs. M. is thirty-six years of age, married, and the mother of two children; the first is now fifteen years of age, the other thirteen.

Her last labor was a difficult one, and after it the patient was attacked with puerperal metro-peritonitis, from which she recovered after a protracted illness of five months duration. Her menses returned at the end of 12 months, but she did not regain her usual vigor under five or six years.

In the early part of the year 1874, she experienced the ordinary symptoms of pregnancy, and believed herself to be in that condition. About three months from the beginning of these symptoms, she noticed bloody discharges from the vagina. They occurred once in three or four weeks. In October, her medical attendant believed the ovum passed away. There were one or two slight discharges after this time. But in contradiction of this opinion, she declared that she felt fœtal movements, and very soon the motion could be felt through the abdominal walls, and by auscultation the beating of the fœtal heart could be distinguished. As time passed, the movements of the fœtus became so painful that she took large doses of morphine for relief. Not long after this practice was begun, the movements of the fœtus ceased. This occurred about three weeks before the time she expected to be confined. The fœtus apparently died Feb. 25, 1875, when Dr. Cowles saw her for the first time, and became the regular attendant; so he found it unnecessary to interfere, and very properly recommended her to wait. He was called again on the 10th of March, and says she presented all the appearances of the first stage of labor. He made an examination of her vagina, and was surprised to find the cervix presenting the conditions of an unimpregnated uterus. He passed his finger into a somewhat patent os uteri, but found no presenting part. He then passed a flexible catheter six inches into the cervix of the uterus, without meeting any obstruction. The patient was afraid that there was no fœtus in that organ, and that delivery was im-impossible—at least in the natural way. In a little time the pains ceased and did not recur; and the patient recovering, resumed her ordinary avocations without much inconvenience.

Things went on in this way until August, 1877. At this time, while walking she came near falling, and after a severe effort to recover herself she felt sharp pains, referred mostly to the left hypochondriac region. General enlargement of the abdomen was noticed soon after this, which has continued to increase up to the present time. The urine became scanty and albuminous, and rapid deterioration of health caused her friends to become alarmed. Her pulse and temperature were both increased, tongue became furred, bowels constipated, and the abdomen, by the middle of October, was increased to twice the size of full-term pregnancy.

The doctor administered diuretics and hydragogue cathartics with decided relief, but he soon became convinced that there was but one way out of the difficulty, and he recommended her to seek relief by coming to Chicago.

She at once assented, and he came with her and, as I have before stated, placed her in the Mercy Hospital, as a last resort.

Now, Gentlemen, this is a pretty clear history of extra-uterine abdominal pregnancy, and if the statements so lucidly given are true, and I see no reason to doubt them, there can hardly be a question of the diagnosis.

Upon making a vaginal examination, I find by the sound the uterus still five inches long, and fixed to the right side of the pelvis. It is very closely connected to a tumor in the right iliac region, plainly perceptible through the abdominal wall at this point. This tumor appears to be very nearly the size of the fœtal head, very hard and only slightly movable. The whole abdomen, as you see, is very much distended with fluid. Fluctuation is very apparent everywhere, except in the region of the tumor, in the iliac region. Upon making sudden and forcible pressure, pressing down the abdominal walls, through this fluid there may be felt a large, irregular, floating body ; there is so much fluid, however, that it is impossible to clearly make out the shape of this body. Near the left hypochondriac region, a round hard substance may be felt, that much resembles the head of a fœtus. Prof. N. S. Davis has examined the patient, and joins me in recommending that an effort be made to relieve the patient by a surgical operation.

The following is an account of the operation which was performed at 2:30 p. m., Nov. 8th, 1877, and of the observations made at the time :

An incision about three inches long in the median line, extending to within about one inch and a half of the symphysis pubis, was made down to the peritoneum. This membrane was very dark colored and extremely vascular. One vein as large as a goose-quill could be seen at the bottom of the incision. After drawing off a small quantity of the fluid by an exploring trochar, the membrane was divided. A great quantity of dark green fluid flowed through the opening. As the abdominal walls collapsed, the irregular outline of the floating mass became very perceptible. The fingers, introduced through the wound, came immediately in contact with a foot of the fœtus, which was drawn out. The incision was then enlarged enough to extract a well-preserved fœtus, which, as was afterwards ascertained, weighed four pounds and eleven ounces. The cord was about two feet long, in a good state of preservation; one end was attached to the umbilicus, and the other was connected to the tumor in the right iliac region, which proved to be the placenta. This last organ was very firm and strongly attached to the uterus, the bladder, and the pelvic brim on the right side, and extended two inches into the iliac fossæ. No attempt was made to remove the placenta, but a strong silk ligature was tied around the cord at its junction with the placenta, and allowed to hang out of the lower end of the wound and the cord divided near it. The fluid was removed from the peritoneal cavity with fine sponges, and the wound closed with silk sutures, as in cases of ovariotomy. The lower end of the wound was left open for three-fourths of an inch for drainage. The external dressing consisted of a thick compress of cotton batting and binder. The patient was then placed in bed and watched until she aroused from the effects of the ether. The operation lasted about twenty minutes.

Two very significant circumstances are mentioned in the history given us by Dr. Cowles. After her last confinement, fifteen years prior to the operation of the extra-uterine pregnancy, the patient suffered with a severe form of metro-peritonitis, from which she recovered after an illness of several months in duration. This inflammation, in all probability, so marred the structure of the Fallopian tube of the right side, as to make it incapable of seizing and transmitting the fructified ovum to the uterus; thus leaving it on or near the ovary, where it found attachment, and became developed into the fœtus and appendages.

The other circumstance I would mention is, the severe and sudden strain to which the abdominal muscles were subjected in the effort the patient made to save herself from falling. From the entire absence of suffering, the want of every disagreeable symptom arising from the presence of the fœtus in the abdominal cavity up to the time of this exertion, and the sudden supervention of peritoneal inflammation, with its consequent effusion of serum, I am led to infer that the fœtus was discharged from its innocuous position at that time. Anyway, it was the date of the commencement of the array of symptoms that induced the disastrous condition afterward realized. From that time the abdominal pain and enlargement can be traced until they eventuated in a broken-down and serious state of health. The appearance of the fœtus, its umbilicus and cord, would also encourage this view; for, as you see, the membranes closely envelop the chord for about two inches from its attachment to the placenta, preserving it in a very remarkable state of freshness. The rest of the cord, up to near its umbilical implantation, is deprived of this extra envelope. Throughout this extent, the cord—which is about twenty inches long—shows signs of maceration. I think that these appearances will fully justify us in concluding that the membranes, while the ovum laid quiescent on the right side, included the whole of the cord coiled up within them, and that when the membranes were torn off from around the umbilicus, it was left floating in the peritoneal fluid and subject to maceration.

Nov. 8, 4 p. m.—Patient rallies from the influence of the ether very slowly; pulse 90, and very feeble; she has vomited several times since the operation.

Eight p. m.—Pulse 78, small and weak; has not vomited since 7 o'clock. The patient has fully recovered her mental faculties, and asks about the results of the operation. At 7:30, she voluntarily passed urine.

Half-past nine, p. m.—Pulse, 78; temperature, 99 2-5°; is resting quietly.

Twelve, midnight.—Urine was drawn with the catheter. The patient is comfortable, and has had a few minutes sleep; pulse, 90, and recovering its strength.

Nov. 9th, 2 a. m.—Pulse, 90; temperature, 99 4-5°. She has had some pain in the abdomen, and feels as though her bowels would move. Up to this time the treatment had consisted of the application of external warmth to the extremities and about the body, and plenty of covering; ¼ grain of the sulphate of morphia was administered hypodermically.

Four a. m.—Has rested well, but has not slept; pulse, 72, still feeble.

Seven a. m.—Pulse, 90 ; temperature, 101 2-5° ; has not vomited since 7 p. m. yesterday, and has taken some beef essence; during the night is very thirsty, and is allowed ice and ice-water.

Half-past ten a. m.—Dressed wound with lint moistened in carbolic oil. There is no discharge from the wound. Pulse, 108, and weak ; tongue moist, and coated with brown fur ; since last visit has taken a teacupful of milk-porridge.

Four p. m.—The patient has just vomited ; pulse, 120, weak and small ; temperature, 101°. Since last visit has had several tablespoonfuls of milk and lime-water, and two ounces of beef essence, containing forty drops of the deodorized tincture of opium.

Half-past seven p. m.—Pulse and temperature same as last visit ; patient vomits frequently the water or other fluid taken ; has pain in abdomen ; an enema of two ounces of beef essence and fifteen drops of deodorized tincture of opium was given. At this visit, thirty minims of brandy were given hypodermically.

At 9:15 a. m.—Gave thirty minims of brandy in same way, and beef essence by enema, in which was dissolved fifteen grains of quinine. The pulse is now 138, very small and weak ; temperature, 101° ; vomiting still continues.

Twelve, midnight.—Pulse, 140, and very weak ; temperature, 101° ; feels comfortable. Beef essence administered, but rejected from the rectum. Hypodermic injection of forty minims of brandy, with one-sixth of a grain of morphia dissolved in it.

Nov. 11, 3 a. m.—Pulse, 140 ; complains of cold feet ; gave an enema of beef essence, fifteen grains quinine, and thirty drops deodorized tincture of opium ; bottles of hot water to the feet.

At 4 a. m., pulse was almost imperceptible, and 150 ; gave thirty minims of brandy hypodermically.

Six a. m., temperature 99°.—The patient says she feels better ; feet and hands are warmer, pulse 130. Introduced the catheter but there was no urine in the bladder.

Nine a. m.—No material change, gave enema of one ounce of beef essence, containing 15 grains quinine. The patient asked for a cup of warm tea, which she drank with a relish. She has some bloody discharge from the vagina.

Twelve m.—No change in pulse and temperature, extremities still somewhat cold. Dressed wound. There is a free discharge of bloody serum from it, 30 minims of brandy saturated with quinine injected hypodermically.

Six p. m.—The patient has vomited several times during the afternoon ; has had champagne several times. Her pulse is 140, temperature 99 3-5. To have 30 minims of saturated solution of quinine in brandy hypodermically, beef essence and quinine by enema as before.

Twelve midnight.—Pulse 130, stronger, temperature 99 3-5 ; still vomits occasionally, has had quinine and brandy hypodermically every two hours, and two enemata of quinine in beef essence. Drew about six ounces of urine. She has rested well during the night, but has not slept. It will hardly be necessary to further give the minutes of treatment in detail. Every effort was made to sustain her strength by hypodermic injections of brandy and quinine, enemata of beef essence, and quinine, brandy and champagne internally as much as the stomach would bear. Waves of elevation and depression of vitality followed each other until 10 p. m. of the 13th, a little over five days after the operation, when she expired.

A post mortem examination was made at 2:30 p. m., Nov. 14th.

The cadaver showed great emaciation and putrescence when the sutures closing the wound were divided. It was ascertained that adhesion had not taken place, and there was no sign of plastic effusion anywhere. The abdominal cavity was freely opened by a large crucial incision. The peritoneal membrane was of a dark livid color, and the sub-peritoneal connective tissue was everywhere occupied by a reticulation of large and small veins. The peritoneal cavity contained about two pints of sero-purulent fluid, and the intestines were collapsed and entirely empty. The placenta was found, as before stated, attached to the right side of the bladder, fundus uteri and pelvis. One margin extended about two inches above the iliopectineal line, and the other about the same distance below it. No trace of the right ovary or Fallopian tube could be found. The placenta was quite solid in structure, and firmly implanted upon the place of attachment, and could not be separated except by dissection. The line of attachment could be easily traced, however, and seemed to be constituted by very dense connective tissue. When separated, it was found to be about one inch and three quarters thick, and four inches in diameter. The fœtal surface was surmounted by a distinct pouch of membranes, that could be lifted up, and which, by the finger introduced in the center, could be explored to the margin of the placenta in its whole circumference. A very short piece of the cord was still attached to the center of the placenta, within the pouch of membranes just described. Upon making an incision into the placenta, numerous minute very red lines, indicating the course of the small arteries, that once constituted the tufts of fœtal vessels, were visible to the naked eye. The bulk of the placenta was transformed into a dense whitish substance, so firm as to require considerable force to break it up.

As before stated, the fœtus had the appearance of being well preserved. The extremities were found to be well formed, in shape and consistence, resembling those of a fœtus expelled but a few hours after its death. The abdomen, chest and head were in no way unusual in appearance. The whole head, body and extremities were covered with the membranes, as with a closely-fitting veil. The membranes adhered to the surface with so much tenacity as to require considerable force to separate them from it. Through these, only the shape of the nose, eyebrows, mouth and chin could be seen. The fingers and toes were covered as with mittens and stockings. The membranes covering the arms and legs surrounded them separately, and did not bind the legs together or pinion the arms to the chest, which seems a singular circumstance. When the membranes were removed from the face and scalp, the features were all salient and distinct, and the hair quite abundant. When the feet and hands were stripped of their covering, the fingers and toes were seen to have been well preserved by their incasement. The skin, too, looked fresh and sound.

Judging from the history of this case, there can be no doubt, I think, that thirty months had transpired from the time of conception, when the operation for its removal was performed. There is also good reason to believe that the fœtus had been dead twenty-two months.

